# Statistical shape analysis of maxillary palatal morphology in patients with palatally displaced canines

**DOI:** 10.1186/s12880-023-01158-4

**Published:** 2023-11-29

**Authors:** Farshad Sobhani, Amirfarhang Miresmaeili, Hossein Mahjub, Maryam Farhadian

**Affiliations:** 1 Department of Biostatistics, School of Public Health, Hamadan University of Medical Sciences, Hamadan, Iran; 2 Department of Orthodontics, School of Dentistry, Hamadan University of Medical Sciences, Hamadan, Iran; 3 Department of Biostatistics, Research Center for Health Sciences, School of Public Health, Hamadan University of Medical Sciences, P.O. Box 4171-65175, Hamadan, Iran

**Keywords:** Palatally displaced canines, Statistical shape analysis, Procrustes analysis

## Abstract

**Objective:**

Maxillary morphology has long been a subject of interest due to its possible impact on palatally and labially displaced canines. This study aims to conduct a comparison of the palate morphology between individuals with palatal and labially displaced canines and control subjects using statistical shape analysis on a coronal cross-sectional of CBCT images.

**Materials and methods:**

Patients aged between 12 and 43 years with palatally or labially displaced canines referred to Hamadan School of Dentistry between 2014 and 2019 were recruited for this retrospective study. The sample included 29 palatally displaced canines (PDC), 20 labially displaced canines (LDC), and 20 control groups (CG). Initially, the maxillary palate coronal section was acquired and landmarked in the region between the right and the left first molar. Procrustes and principal component analyses were used to identify the primary patterns of palatal shape variation. Statistical tests were then performed to examine both shape and size differences.

**Results:**

According to the results of Hotelling’s T2 test, there is a significant difference between the mean shape of palate in PDC and CG (*P* = 0.009), while the difference between the PDC-LDC and LDC-CG groups is not significant. The longest full Procrustes distance was observed between PDC and CG (distance = 0.043), and the shortest full Procrustes distance was observed between LDC and CG (distance = 0.029). The first two principal components accounted for 84.47% of the total variance. The predictive accuracy of the discriminant analysis model showed that 72.46% of cases were correctly classified into the three study groups.

**Conclusions:**

In terms of centroid size, there was no significant difference in the sectional area between the three groups, but the difference between the mean shape of palate in the PDC and CG groups was significant. The PDC group showed more prominent mid-palatal area in the molar region.

## Introduction

Palatally impacted canines are a commonly occurring tooth displacement that can lead to various complications. When teeth are impacted, it can result in adjacent teeth experiencing root resorption or displacement, and a reduction in the width and length of the dental arch can occur. Further problems can arise, including malocclusion and cysts on the affected tooth [1, 2]. Moreover, it is important to consider that surgically exposing an impacted tooth and realigning it within the dental arch for proper positioning requires lengthy treatment duration and significant financial expenses [3, 4].

The prevalence of impacted canines is higher than that of other teeth after the third molar. In addition, it has been reported that the prevalence of upper canines is 10 to 20 times more frequent than that of lower canines. Impacted maxillary canines are located on the palatal side in 85% of cases and on the buccal side in 15% of cases [5–7]. Although some reports suggest that the direction of incidence varies by ethnicity. Female-to-male prevalence ratios vary from 1.3:1 through 3.2:1 [8].

Several hypotheses have been proposed regarding the etiology of impacted canines. However, the results of various studies are controversial and the reasons for impaction remain unclear [9, 10].

Maxillary morphology has always attracted much attention as one of the influencing factors in the development of palatally and/or labially displaced canines. The occurrence of impacted canines and the skeletal and dental dimensions of the maxilla have been investigated by several investigators. Linear measurements in patients with palatally displaced canines have been compared in many studies [5–7]. Some studies suggest that a lack of maxillary width may mechanically cause impacted canines, while others report no such association. Although linear measurements cannot easily summarize the complex shape of an area [11–16].

Also, many studies have used dental casts to assess maxillary width, but these measurements are prone to bias because changes calculated by conventional methods may not always reflect the modified arch configuration. Linear measurements made on dental casts or digital models can be affected by the inclination of the tooth and variable thickness of soft tissue, which can lead to inaccuracies. Therefore, the use of cone beam computed tomography (CBCT) now facilitates obtaining accurate information about bone dimensions by providing high-resolution three-dimensional images of teeth and bone [17–19].

Linear measurements consist of length, depth, and width, which do not provide significant information on the geometry of the structure being investigated. While it can be difficult to formally distinguish between size and shape in the traditional framework. Recent advancements in geometric morphometrics have produced novel techniques for precisely measuring size and shape. It also has the ability to visualize morphological differences. Morphometric methods mainly include two categories of methods: traditional morphometric methods based on statistical analysis of distances measured on the biological structure, such as length, width, depth, and sometimes ratios and angles, etc. Geometric morphometric methods are based on landmarks and semi-landmarks (i.e., a point on a curve) to obtain geometric information from biological structures [20, 21].

The combination of geometric morphometric techniques with multivariate statistical techniques is a highly effective tool for the investigation and visualization of shape differences. Landmark-based methods involve comparisons between shapes and forms based on information from two-dimensional (x, y) or three-dimensional (x, y, z) landmark points as homologous points [22].

Among the advantages of this method is the possibility to keep the geometric position of the landmarks during the analysis and to present the graphical results in the form of deformation grids and a simple interpretation of the graphs compared to tables with numerical coefficients [21]. Statistical shape analysis can be used in various fields such as forensics, biology, genetics, archaeology, etc. [23, 24].

To our knowledge, no prior research has employed statistical shape analysis to examine maxillary palate morphology in cases of displaced canines and compared it with control samples. The aim of this study was to investigate the morphology of the maxillary palate in patients with palatally/labially impacted canines using shape analysis based on a cross-section of CBCT images and to compare it with the control group at the level of the first molar. A discriminant model is also presented, which predicts impacted canines based on the size and shape of the palate in the maxillary region.

## Materials and methods

This study was a retrospective analytical study of 69 patients aged 12 to 43 years who were referred to Hamadan School of Dentistry with palatally and labially displaced canines between 2014 and 2019. They were divided into 3 groups: the palatally displaced canines (PDC = 29), the labially displaced canines (LDC = 20), and the control group (CG = 20). The CG was subjects whose canine had grown and did not have palatal constrictions. Ethical approval for the study was obtained from the Ethics Committee of Hamadan University of Medical Sciences (IR.UMSHA.REC. 1397.1034).

### Inclusion and exclusion criteria

This study included patients aged 12 years and older who had CBCT images of the maxilla with labial or palatal canine impaction.

Patients who have previously undergone orthodontic treatment, those with confirmed obstructions such as odontoma or supernumerary teeth, individuals with systemic illnesses, those with superfluous teeth, patients who have had facial bone or soft tissue surgery, those with craniofacial anomalies like cleft lip or palate, and those with harmful oral habits like thumb-sucking, tongue-thrusting, and mouth-breathing, as well as those with multiple impacted teeth or congenitally missing teeth, are excluded from this study. The CBCT images of these individuals were obtained for reasons such as implant placement, trauma, or diagnostic reasons. In addition, patients in the two or three groups were matched for age and gender.

### Image preparation

Participants’ CBCT images were acquired using the NewTom 3G device (QR-DVT9000, Versona, Italy), Dicom-format images were transferred to Dolphin version 11.5 (Chatsworth, California) to prepare the coronal cross section from inter 1^st^ molar region.

### Landmarking with tpsDig2.31

**Fig. 1:**
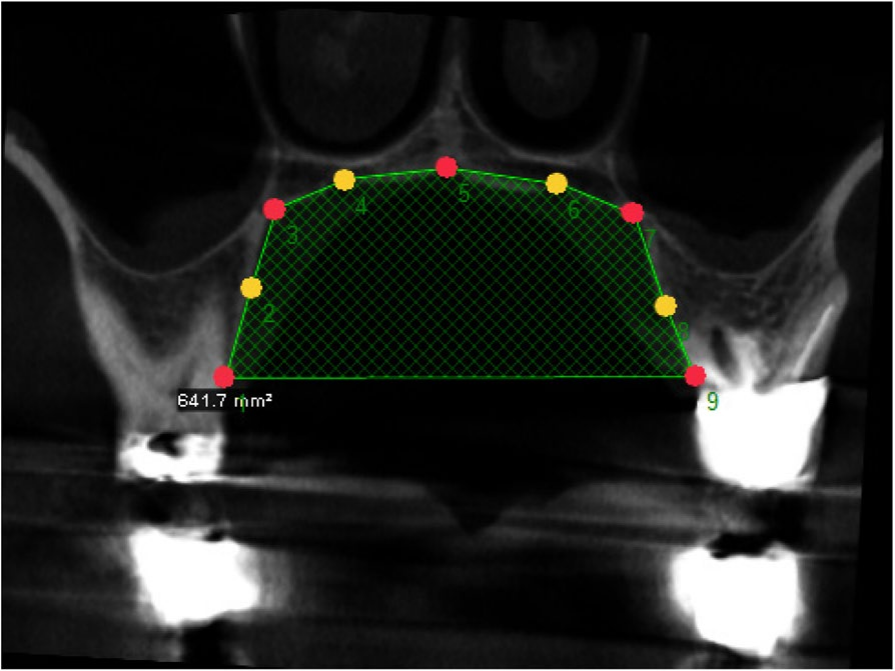
Position of 9 landmarks (5 main points in red and 4 supplementary points in yellow) on a case of the CG: edge of the alveolar ridge on the right (point 9) and left (point 1), the junction of the vertical wall of the alveoli with the roof of the mouth on the right (point 8) and left (point 2), and the middle palatal suture (point 5). Complementary points 2, 4, 6 and 8 are the midpoints between the five main points mentioned above

In the first step, the 3D images were transferred to the Dolphin software by an orthodontist and standardized according to the palatal plane. Subsequently, a coronal section was made in the area of the first maxillary molars and the position of the landmarks in this section was determined. The landmarks determined consisted of 5 main points, including the edge of the alveolar ridge on the right (landmark 9) and left (landmark 1), the junction of the vertical wall of the alveoli with the roof of the mouth on the right (landmark 8) and left (landmark 2), and the midpoint of the palatal suture (landmark 5). The four additional points 2, 4, 6, and 8 are the midpoints between the above five main points (Fig. 1).

### Statistical shape analysis steps

After determining the position of the landmarks on the images with the software tpsDig2.31, digitization was performed with the software R version 3.6.0 (Shapes and Morpho packages). Then, the two-dimensional coordinate matrix (x–y matrix with dimensions 9*2) is extracted.

Subsequently, the Procrustes superimposition was assessed based on the Cartesian coordinates generated by the landmarks. This method converts the coordinates of the original data to shape coordinates. Generalized Procrust analysis was performed with the aim of removing non-shape differences such as direction, position, and scale, as well as superimposing the landmarks of the samples.

To compare the size of the studied groups, the centroid size was used. The centroid size is the squared root of the sum of squared distances between all landmarks and their centroid, i.e., it is the arithmetic mean of all landmarks. Due to centroid size being uncorrelated with shape, any shape described by the same number of landmarks may be compared in terms of its size using the centroid size index [23].

Also, Procrustes distances were calculated to check the variation in the shape of the assessed groups. Procrustes distance is the square root of the sum of squared differences in the position of the landmarks in two shapes. This can be used to describe the difference between many landmark configurations [23].

Once generalized Procrustes analysis is performed on landmark configurations, a mean shape configuration (consensus) is calculated and variation around this mean can be decomposed into components of morphological variation. Shape spaces are curved and a projection onto a tangent space with the consensus as the point of tangency is used to create a shape tangent space. In this shape tangent space, conventional Euclidean statistical methods are viable, such as Principal Component Analysis (PCA). PCA was applied to give insight into the average shape and shape variation of the data set. Principal component scores are “shape variables” that are the basis for further analysis.

Visualization of palate shape changes based on the average shape of the population compared to the overall average shape (Consensus configuration) was done using the deformation grid plot.

A model for predicting impacted canines based on the shape and size of the palate in the maxillary region is also presented. The shape-coordinate matrix obtained from the Procrustes analysis was used as the input variable in the discriminant analysis. It should be noted that the information related to the shape variables is in the extracted principal components.

In order to evaluate the performance of the prediction model, the confusion matrix was used. A Confusion matrix is an N x N matrix used for evaluating the performance of a predictive model, where N is the number of target classes (3 classes in this study). The matrix compares the actual target values with those predicted by the discriminant model.

To determine the accuracy of a discriminant model using the confusion matrix, the number of correctly predicted samples is summed, and the resulting sum is divided by the total number of samples.

## Results

Of the 69 subjects, 20 were CG (11 females and 9 males), 29 were PDC (17 females and 12 males), and 20 were LDC (11 females and 9 males).

### Reproducibility assessment using the protest

In order to ensure the reproducibility of the results (digitization of the landmarks), the procedure for determining the position of the landmarks was performed in two repetitions by an experienced orthodontist (Fig. 1). In this way, the position of the landmarks was determined for all images in two repetitions and a two-dimensional matrix was extracted from the shape information. The results of applying the Protest in 1000 repetitions confirm the repeatability of the results (*p* < 0.001).

### Group comparison in terms of maxillary palate size

The results of the t-test show that there is no statistically significant difference between the two groups in terms of centralized size. However, the displaced maxillary canines (DMC) patients tend to have larger maxillary palate size than those in the control group (Table 1).

**Table 1:** Comparison of groups in terms of centroid size

Group	Mean ± SD	*P*-value^*^
PDC	225.24 ± 35.92	0.573
LDC	219.16 ± 38.07	
PDC	225.24 ± 35.92	0.682
CG	213.14 ± 53	
LDC	219.16 ± 38.07	0.345
CG	213.14 ± 53	

*PDC* Palatally displaced canines, *LDC* Labially displaced canines, *CG* Control group

^*^t-test

Regarding the gender comparison, although centroid sizes were greater in males than in females, the mean size area between both genders was not significant (Table 2).

**Table 2:** Comparison of groups in terms of centroid size by genders

Group	Sex	Mean ± SD	*P*-value^*^
PDC	Male	251.64 ± 23.91	0.061
	Female	223.31 ± 38.90	
LDC	Male	224.02 ± 43.24	0.916
	Female	221.97 ± 41.89	
CG	Male	218.61 ± 36.65	0.671
	Female	208.66 ± 64.91	

*PDC* Palatally displaced canines, *LDC* Labially displaced canines, *CG* Control group

^*^t-test

### Group comparison in terms of maxillary palate shape

According to the results of the Hotelling T2 test based on the bootstrap samples for comparing the shape variables (Table 3); a statistically significant difference was found between the shape of the PDC and CG groups. After combining the PDC and LCD groups with respect to the group of impacted canines, the results showed that there was also a statistically significant difference compared with the CG group. These findings are confirmed by the results of the permutation test which also compare the mean shape difference.

**Table 3:** Comparison of groups in terms of mean shape using permutation and bootstrap tests

Group	*P*-value^*^	*P*-value^**^
PDC – CG	**0.017**	**0.009**
LDC – CG	0.155	0.108
PDC – LDC	0.087	0.158
Control – (PDC + LDC)	**0.002**	**0.009**

*PDC* Palatally displaced canines, *LDC* Labially displaced canines, *CG* Control group

^*^Hotelling T2 test (bootstrap)

^**^Hotelling T2 test (permutation)

Also, the highest Procrustes distance was observed in the PDC and CG groups (0.043), while the lowest distance was observed between the LDC and CG groups (0.029). The Procrustes distance between the PDC and LDC groups was 0.034.

### Development of a prediction model for displaced canines based on the morphology of the maxillary palate

The first and second principal components explained 75.68% and 8.79% of the shape variance respectively. Table 4 shows that the discriminant analysis model based on the first two principal components is able to correctly classify people into the three study groups with a prediction accuracy of 72.46%. The predictive ability of this model is 80% for the LDC group (i.e. 16 out of 20), 68.9% for the PDC group (i.e. 20 out of 29) and 70% (i.e. 14 out of 20) for the control group.

**Table 4:** Confusion matrix for prediction of impacted canine based on discriminant analysis

		**True**					
		LDC	PDC	CG	Total	%Correct	Total Accuracy
**Predict**	LDC	16	5	3	24	80%	72.46%
	PDC	2	20	3	25	68.9%	
	CG	2	4	14	20	70%	
	Total	20	29	20	69		

*PDC* Palatally displaced canines, *LDC* Labially displaced canines, *CG* Control group

### Visualization of maxillary palate morphology

**Fig. 2:**
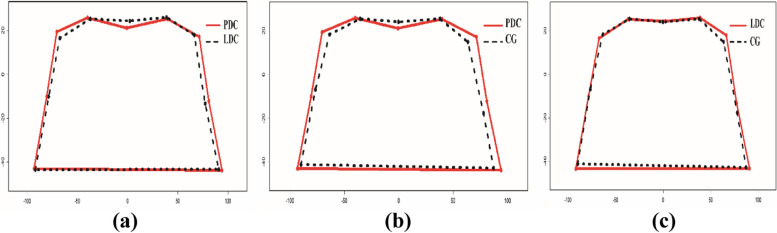
Schematic comparison of the mean shape with the generalized Procrustes method: **a** Schematic comparison between the mean shape of the LDC and PDC groups. **b** Schematic comparison between the mean shape of the CG and the PDC group. **c** Schematic comparison between the mean shape of the CG and the LDC group

Figure 2 also shows a schematic comparison of the mean shape of study groups with the generalized Procrustes method. It can be seen that the maxillary region in the PDC group shows greater elongation compared to the other two groups. Also, the transverse dimension of the study area appears to be slightly wider in patients with PDC.

**Fig. 3:**
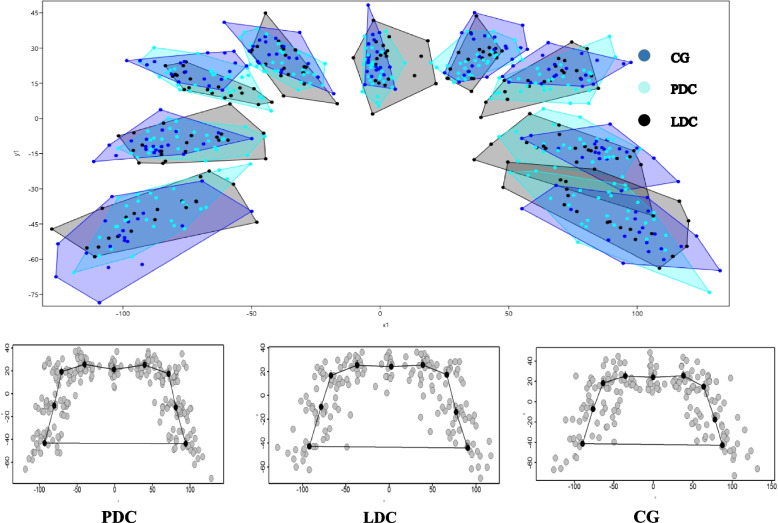
Variation of each group according to nine landmarks. PDC: Palatally displaced canines; LDC: Labially displaced canines; CG: Control group

Figure 3 displays the landmark variations for each of the studied groups separately. Furthermore, the landmark variations for all subjects were collectively presented alongside the group averages. In the PDC group, the palatal area at landmark No. 5 displays a heart-shaped downward curve, unlike the labial and control groups where this region appears similar and relatively flat. The variation in distribution increases as we move towards the sides from the center. The landmark displaying the greatest variation in all groups is #1 (the edge of the left alveolar ridge) and #9 (the edge of the right alveolar ridge).

## Discussion

In this study, statistical shape analysis was used to investigate the morphology of the maxillary palate in the molar region of samples with impacted canines. Although there was no statistically significant difference between the groups regarding centroid size, a significant difference in the shape was observed between the PDC and CG groups, as well as the group resulting from the combination of PDC and LDC in comparison to CG. In addition, the resultant graphical image is confirmed that midline of the plate in subjects with palatally impacted exhibited a heart-shaped form in comparison with the labial impaction and control groups. However, further studies are recommended to establish the clinical applicability of present finding. Morphological analysis of the palate in PDC could help dentists find a positive sign of impaction when the canine has not appeared in the mouth at the correct time especially before radiographic examination.

A comparison of the shape parameters and the associated graphs verify that the maxillary region is wider in the PDC group compared to the other two groups. Various studies regarding the width of the maxillary arch and the presence of impacted canines show different results. McConnell et al. suggest that palatal canines exhibit insufficient maxillary arch width, while some studies show that this relationship is not significant [11]. A study examining the Linear measurements in maxillary arch between PDC and LDC revealed that although there is not significant differences in larch length and intermolar distance the ratio of arch length to intermolar distance is greater in PDC cases relative to LDC [5, 9].

Limited studies used the statistical shape method to evaluate the morphology of the maxillary region in patients with impacted canines; Mucedero et al. used a three-dimensional geometric morphometric method. The results of comparing the groups (one and two impacted canines compared with CG) showed no significant difference in palatal shape. However, the width between the canines of t’he impacted groups based on linear measurements was significant compared to the CG subjects [13].

Most studies were conducted using traditional morphological methods based on linear measurements. For example, Kim et al. conducted a study to investigate the relationship between the position of impacted canines and maxillary morphology. The ratio of palatal vault depth/intermolar width and arch length/intermolar width was used to compare the shapes of the palatal arch and maxillary arch. The results showed that no significant difference between intermolar distance in PDC and LDC, the shape of the maxillary arch was more flat “U shaped” and with deeper palatal vault in the PDC than LDC [5]. Another study showed that while intermolar distance increased at 1^st^ molar region, due to reduced palatal vault depth total area in coronal sectional area reduced in PDC relative to LDC [4]. In the present study, according to the graphical results and the registering landmarks, the palatal arch was wider in the PDC, and the midline of the palatal suture (landmark 5) was also curved downward compared with the LDC and CG.

Miresmaeili et al. studied the morphology of the maxilla in patients with unilaterally and bilaterally palatally displaced canines and control groups using the linear and area measurements. The results of this study showed that there was a statistically significant difference in palatal intermolar area and depth of palatal vault between the groups. They found a decrease in palatal intermolar area and depth of palatal vault tended to correlate with palatal displacement of the canines [25].

Fattahi et al. investigated indices such as palatal height, maxillary arch intermolar width, and maxillary arch length between the impacted and non-impacted sides in an Iranian population. The results of this study show that maxillary arc length is the only indicator that has a statistically lower in impacted canine group while the other variables are similar in both groups [26]. So the linear and area measurement analysis cannot show a consistent results.

The results of the present study showed that the centroid size of males was larger than in females in the three groups, although this difference was not significant. Similar findings regarding a significant increase in intercanine and intermolar width in male compared to female have been observed in other studies [27, 28].

Saade et al., conducted a study of maxillary dimensions and arch shape based on the CBCT images to compare occlusal and skeletal measurements between PDC and control. The results confirmed that the skeletal measurements were generally larger in the PDC group, but the transversal measurements were not significant. Discriminant analysis also showed an accurate classification of 85.9% for the control group and 66.7% for the PDC group. In the present study, prediction accuracy was 70% for the control group and 68.9% for the PDC [29].

In addition to demographic differences, these different results may be due to different measurement methods and tools, such as CBCT and dental casts. Therefore, further research in this area is proposed, based on statistical shape analysis with the ability to preserve the size and shape of the areas of interest in order to draw more reliable conclusions.

In the present study, two-dimensional landmarks were used to analyze the data. For future studies, the use of three-dimensional landmarks is suggested, and the use of additional supplementary landmarks could provide more comprehensive information about the morphology of the maxillary regions of impacted teeth. In addition, it is recommended to evaluate the shape of maxillary regions in cross-section of other teeth and also buccal alveolar bone in incisor region.

## Conclusion

In terms of centroid size, there was no significant difference in the sectional area between the three groups, but the difference between the mean shape of the PDC and CG groups was significant. Because the midline of the palate is relatively prominent in patients with palatally displaced canines, touching and observing this area can be used as a clinical sign to predict impacted teeth.

## Data Availability

The datasets generated and/or analyzed in the current study are available upon request from the corresponding author.

## Abbreviations

PDC: Palatally displaced canines
LDC: Labially displaced canines
CG: Control group
CBCT: Cone beam computed tomography
